# Diagnostic performance of choline PET/CT for the detection of bone metastasis in prostate cancer: A systematic review and meta-analysis

**DOI:** 10.1371/journal.pone.0203400

**Published:** 2018-09-07

**Authors:** Yu Guo, Ling Wang, Junjie Hu, Dehong Feng, Lijuan Xu

**Affiliations:** 1 Department of Orthopaedic Surgery, The Affiliated Wuxi People's Hospital of Nanjing Medical University, Wuxi, Jiangsu, China; 2 Department of Orthopaedic Surgery, Suzhou Dushuhu Public Hospital (Soochow University Multi-Disciplinary Polyclinic), Suzhou, Jiangsu, China; 3 Department of Urology, The Affiliated Wuxi People's Hospital of Nanjing Medical University, Wuxi, Jiangsu, China; 4 Laboratory Center, The Affiliated Suzhou Hospital of Nanjing Medical University, The North District of Suzhou Municipal Hospital, Suzhou, Jiangsu, China; Ente Ospedaliero Cantonale, SWITZERLAND

## Abstract

**Purpose:**

The aim of this study was to evaluate the diagnostic performance of choline positron emission tomography/computed tomography (PET/CT) for the detection of bone metastasis in patients with prostate cancer.

**Methods:**

MEDLINE, EMBASE and the Cochrane Library were searched up to 20 February 2018 for studies that used 11C-choline or 18F-choline PET/CT for the detection of bone metastasis in patients with prostate cancer and “histopathology and/or clinical follow-up” as the reference standard. Methodological quality was assessed using the Quality Assessment of Diagnostic Accuracy Studies-2 (QUADAS-2) tool. Pooled diagnostic accuracy with the 95% confidence interval (CI) was calculated using a bivariate random effects model. We also constructed hierarchical summary receiver operating characteristic curves and performed meta-regression analyses.

**Results:**

Fourteen studies with reasonable methodological quality were included in the analysis. On a per-patient basis, the pooled sensitivity, specificity, positive likelihood ratio (PLR), negative likelihood ratio (NLR), and diagnostic odds ratio (DOR) were 0.89 (95% CI 0.80–0.94), 0.98 (95% CI 0.95–0.99), 40.4 (95% CI 19.7–82.6), 0.12 (95% CI 0.07–0.20), and 344 (95% CI 148–803), respectively. On a per-lesion basis, the pooled sensitivity, specificity, PLR, NLR, and DOR were 0.91 (95% CI 0.85–0.94), 0.97 (95% CI 0.95–0.98), 34.1 (95% CI 20.0–58.1), 0.10 (95% CI 0.06–0.16), and 358 (95% CI 165–778), respectively. In the meta-regression analysis, the clinical setting (staging vs. restaging) was the only source of study heterogeneity on a per-patient basis.

**Conclusions:**

Choline PET/CT shows excellent diagnostic performance for the detection of bone metastasis. However, a negative choline PET/CT result cannot ensure the lack of bone metastasis.

## Introduction

Prostate cancer (PC) is the second most common malignancy in males worldwide, with an incidence of approximately 1.1 million cases per year [1, 2]. Bone and lymph nodes are common sites of metastasis in PC [3]. Approximately 8–35% of PC patients at initial diagnosis and 65–75% of advanced PC patients will develop bone metastases [4–6]. It is universally acknowledged that skeletal metastases are a major cause of death, disability, and that they decrease quality of life and increase the cost of treatment [7, 8]. Therefore, it is critically important to accurately detect bone metastasis in order to select appropriate patient management.

Currently, most institutions use conventional imaging modalities such as bone scan (BS), computed tomography (CT) and magnetic resonance imaging (MRI) to detect bone metastases of PC. However, conventional modalities are not always reliable in the evaluation of metastatic bone lesions [3, 9]. Therefore, a new imaging technique is needed for improving diagnostic performance. In recent decades, integrated PET/CT has emerged as a new modality for whole-body imaging; this modality provides both the metabolic processes and comprehensive morphological information in a single examination [10]. 18F-fluorodeoxyglucose (18F-FDG) is the most widely used tracer; however, it has limited value for clinical imaging in PC due to the low glucose metabolism and confounding influence of bladder activity [11]. As alternatives to 18F-FDG, more recent radiotracers, such as 11C-choline and 18F-choline, have shown promising results [12–14]. Thus far, although there have been many studies investigating choline PET/CT for the diagnosis of bone metastases in PC patients, the results from these studies remain inconsistent.

Up to date, only two meta-analyses have investigated the accuracy of choline PET/CT for the diagnosis of skeletal metastases in PC with conflicting results and methodological limitation [15, 16]. Since the publication of the last meta-analyses, new diagnostic studies mostly with satisfactory methodology have been performed. Additionally, the bivariate random effects regression model has been proposed to optimize diagnostic meta-analysis [17]. This statistical approach estimates pairs of logit transformed sensitivity and specificity of a diagnostic test and provides more precise estimates of the diagnostic accuracy.

Accordingly, we conducted a systematic review and meta-analysis to evaluate the diagnostic performance of 11C-choline and 18F-choline PET/CT for the detection of bone metastasis in PC patients.

## Methods

### Literature search

This systematic review and meta-analysis was conducted in accordance with the Preferred Reporting Items for Systematic Reviews and Meta-Analysis (PRISMA) guidelines. We systematically searched the Medline, Embase and the Cochrane Library databases up to 20 February 2018. The following search terms were used: ([‘‘prostat* cancer”] OR [‘‘prostat* carcinoma”] OR [‘‘prostat* neoplasm”] OR [‘‘prostat* tumor”]) AND ([choline] OR [18F-choline] OR [18F-FCH] OR [11C-choline] OR [fluorocholine] OR [FCH]) AND ([‘‘positron emission tomography computed tomography”] OR [PET/CT] OR [‘‘positron emission tomography”] OR [PET]) AND ([bone] OR [skeletal]). We also manually searched other relevant references to identify potential articles.

### Study selection

Two investigators independently screened titles and abstracts of all citations. Discrepancies were resolved by mutual agreement. We then reviewed the full text of these studies deemed relevant to determine eligibility. Studies were included based on the following criteria: (1) patients diagnosed with PC regardless of disease stage and treatment status, (2) 11C-choline or 18F-choline PET/CT used as the index test for detecting bone metastasis, (3) sufficient data to construct 2×2 contingency tables regarding sensitivity and specificity, (4) histopathological results and/or clinical follow-up served as the reference standard, and (5) publications written in English. The exclusion criteria were as follows: (1) case series with fewer than 10 patients; (2) insufficient data to construct contingency tables; (3) duplicated studies enrolling the same cohort; and (4) reviews, conference abstracts, case reports, and letters. In the case of an overlapping population, only the largest and most informative study was included.

### Data extraction and quality assessment

Two investigators independently performed data extraction, and disagreements were resolved by consensus or consultation with a third reviewer. The following information was extracted using a standardized form: authors, publication year, country, study design, reference standard, blinding to reference standard, patient characteristics, clinical setting, prostate specific antigen (PSA) level, PET/CT characteristics, and absolute number of true positive, false positive, true negative, and false negative results for either patient-based analysis or lesion-based analysis. The authors of the eligible studies with inadequate data were contacted through email for additional information.

The quality of each study was independently appraised by two observers using the QUADAS-2 tool [18]. The QUADAS-2 tool assesses the risk of bias and applicability based on four domains: patient selection, index text, reference standard, and flow and timing. The provided signaling questions of the QUADAS-2 tool were used to reach a judgment as ‘low’, ‘high’, or ‘unclear’ rating.

### Statistical analysis

The pooled summary estimates of sensitivity, specificity, positive likelihood ratio (PLR), negative likelihood ratio (NLR), and diagnostic odds ratio (DOR) were calculated. We used a bivariate random effects regression approach to synthesize data. This method estimated pairs of logit transformed sensitivities and specificities from studies following a bivariate normal distribution, incorporating both the between-study and within-study variability. Summary estimates of sensitivity and specificity were plotted in forest plots and hierarchical summary receiver operating characteristic (HSROC) curves with 95% confidence and prediction regions. A Spearman correlation coefficient of greater than 0.6 was considered to indicate a considerable threshold effect. Deeks’ funnel plot was conducted to detect publication bias [19].

Heterogeneity was assessed using Cochran’s Q test (p < 0.05 was considered significant) and the I^2^ index (I^2^ > 50% was considered substantial heterogeneity) [20]. We performed meta-regression to investigate the potential source of heterogeneity within the included studies. The covariates included in the analysis were as follows: study design (prospective vs. retrospective), tracer (11C-choline vs. 18F-choline), clinical setting (staging vs. restaging), reference standard (histopathology or clinical follow-up vs. only clinical follow-up), diagnostic criteria (qualitative and semi-quantitative vs. qualitative), and blinding to reference standard (yes vs. no). Statistical analyses were performed using STATA version 12.0 (STATA Corporation, College Station, TX, USA). The association was considered statistically significant if the p value was less than 0.05.

## Results

### Eligible studies and study description

**Fig 1:**
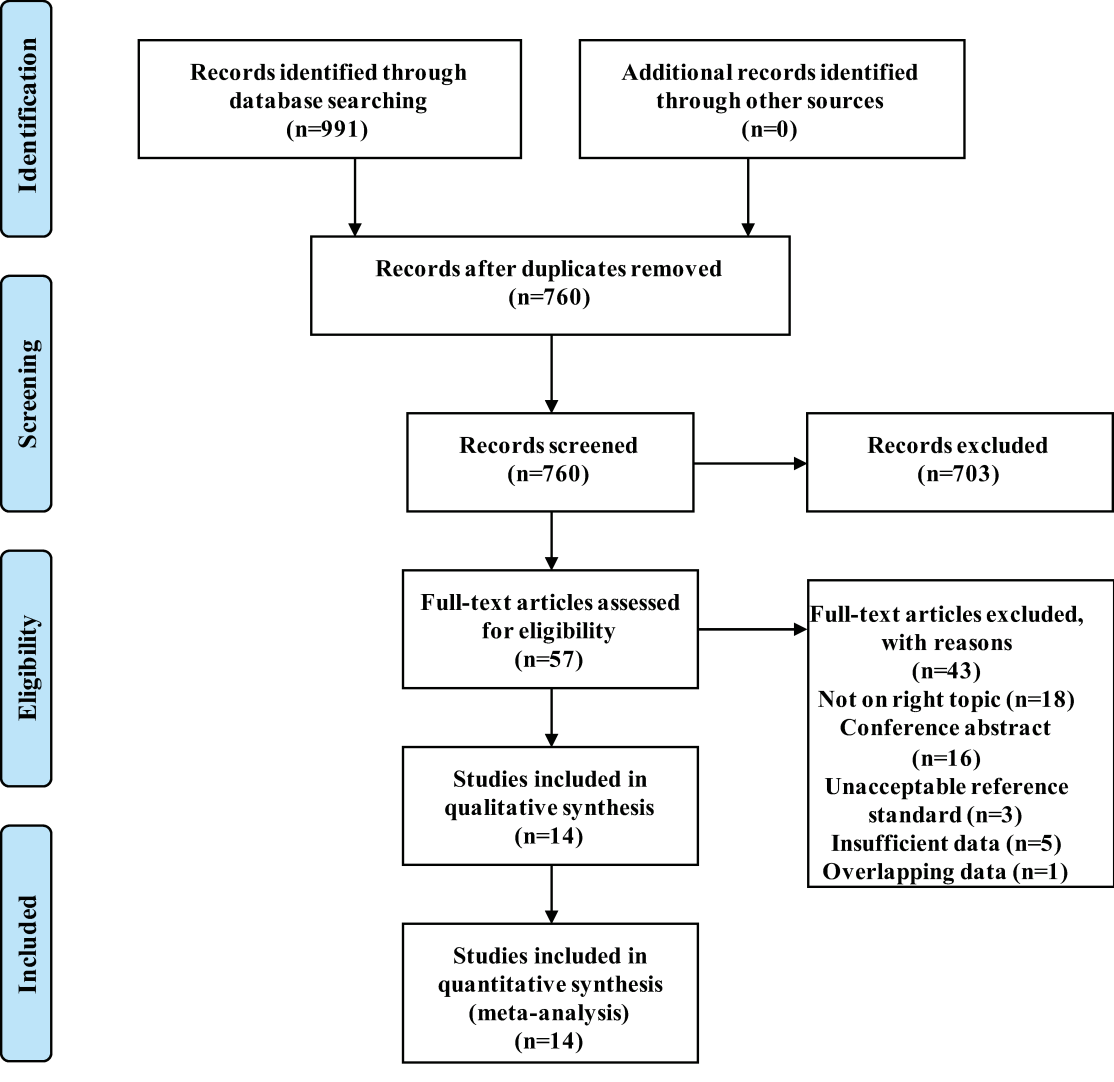
Flow diagram describing the process of the systematic search and selection.

The process of study selection is shown in Fig 1. The systematic search retrieved 760 articles after removing 231 duplicates. Among these articles, 57 articles were selected for reading of the full text. Finally, 14 studies [12–14, 21–31] were included based on the inclusion and exclusion criteria. The general study characteristics are presented in Table 1. Six studies were analyzed on a per-patient basis, four studies were analyzed on a per-lesion basis, and four studies were analyzed on a per-patient basis as well as per-lesion basis. Five studies used “histopathology and/or clinical follow-up” as the reference standard, while the other nine used only clinical follow-up. The PET/CT characteristics are shown in Table 2. 11C-choline as a tracer was used in seven studies, and 18F-choline was used in the other seven studies. There was a wide variation in imaging protocols, particularly regarding the injection dose of tracers and the time from injection to scan.

**Table 1:** Characteristics of the included studies.

Study references	Country	Design	Clinical setting	Number of patients	Age^a^	Analysis	PSA (ng/ml)			Reference standard	Previous therapy
							Mean	Median	Range		
Beheshti et al, 2010 [12]	Austria	Pro	Staging and restaging	70	68±7	L	39.65	NR	0.1–239	Clinical follow-up	None, RP, RT, HT, chemotherapy
Evangelista et al, 2015 [22]	Italy	Retro	Staging	48	70±9 (49–86)	P	38.34 ± 90.12	12.7	2.80–581.0	Clinical follow-up	None
Fuccio et al, 2010 [13]	Italy	Retro	Restaging	25	70.2 (58–80)	P, L	11.1 ± 14.38	6.3	0.2–37.7	Histopathology and/or clinical follow-up	RP, partial prostatectomy, RT
Garcia et al, 2015 [27]	Spain	Pro	Restaging	169	65±11	P, L	4.8	NR	2.4–58	Histopathology and/or clinical follow-up	Prostatectomy, RT
Huysse et al, 2017 [28]	Belgium	Pro	Restaging	64	NR	P, L	NR	3.1	1.2–6.5	Clinical follow-up	RP, RT, ADT
Kitajima et al, 2014 [23]	America	Retro	Restaging	95	65.7 (49–87)	P	5.26	2.5	0.58–68.3	Histopathology and/or clinical follow-up	RP, salvage EBRT, ADT, salvage cryoablation
Kitajima et al, 2017 [26]	Japan	Pro	Staging and restaging	21	70.6±10.8 (47–90)	P	342.9	NR	0.2–5916	Clinical follow-up	None, Prostatectomy, RT, HT
Langsteger et al, 2011 [14]	France, Austria	Pro	Staging and restaging	40	66 (51–82)	P, L	NR	NR	0.38–617	Clinical follow-up	NR
McCarthy et al, 2011 [29]	Australia	Pro	Restaging	26	75.4±8.4 (62–89)	L	NR	10.5	1.6–250	Clinical follow-up	NR
Nanni et al, 2016 [25]	Italy	Pro	Restaging	89	69 (55–83)	P	6.99±17.5	3.35	0.20–20.72	Histopathology and/or clinical follow-up	RP, RT, HT
Picchio et al, 2012 [21]	Italy	Retro	Restaging	78	69 (47–82)	P	21.1	2.4	0.2–500	Clinical follow-up	RP, RT, HT
Piccardo et al, 2014 [30]	Italy	Pro	Restaging	21	77.2±5.1 (70–85)	L	5.8 ± 3.4	4.9	2.2–13.4	Clinical follow-up	EBRT, ADT
Takesh et al, 2012 [24]	Germany	Retro	Restaging	37	69±7	P	NR	2.6	0.3–21	Clinical follow-up	RP, RT, HT, ADT
Wieder et al, 2017 [31]	Germany	Pro	Restaging	57	68 (54–80)	L	29.9	NR	1.0–670	Histopathology and/or clinical follow-up	RP

^a^Expressed as median or mean ± standard deviation (range)

Pro, prospective; Retro, retrospective; L lesion-based; P, patient-based; PSA, prostate-specific antigen; RP, radical prostatectomy; RT, radiotherapy; HT, hormone therapy; ADT, androgen deprivation therapy; EBRT, external beam radiotherapy; NR, not reported.

**Table 2:** Characteristics of PET/CT.

Study references	Tracer	Injection Dose	Imaging analysis	Time^a^ (min)	Interpreters	Blinding	Manufacture	Follow-up (months)
Beheshti et al, 2010 [12]	18F	4.07 MBq/kg	QL, Semi-QN	1	3	N	Discovery LS (GE Medical Systems)	6–15
Evangelista et al, 2015 [22]	18F	3 MBq/kg	QL	60	2	NR	Siemens Biograph(Siemens)	>6
Fuccio et al, 2010 [13]	11C	370–555 MBq	QL	5	2	NR	Discovery LS (GE Healthcare)	>6
Garcia et al, 2015 [27]	11C	296 MBq	QL, Semi-QN	5	2	Y	NR	6–15
Huysse et al, 2017 [28]	18F	3–4 MBq/kg	NR	45	2	Y	NR	NR
Kitajima et al, 2014 [23]	11C	370–555 MBq	QL, Semi-QN	5	2	Y	Discovery RX or Discovery 690 (GE Healthcare)	>6
Kitajima et al, 2017 [26]	11C	3 MBq/kg	QL, Semi-QN	5	2	Y	Gemini TF64 (Philips Medical Systems)	NR
Langsteger et al, 2011 [14]	18F	4 MBq/kg	NR	10–20	2	Y	Discovery LS (GE Medical Systems) or Gemini Dual (Philips)	>6
McCarthy et al, 2011 [29]	18F	200 MBq	QL, Semi-QN	5	2	Y	GSO (Philips Allegro) or Siemens Biograph (Siemens)	6–14
Nanni et al, 2016 [25]	11C	3.4 MBq/kg	QL, Semi-QN	3–5	2	NR	Discovery STE (GE Healthcare)	6–29
Picchio et al, 2012 [21]	11C	370 MBq	QL	5	2	Y	Discovery LS, Discovery ST or Discovery STE scanner (GE Medical Systems)	18 (mean)
Piccardo et al, 2014 [30]	18F	3 MBq/kg	QL, Semi-QN	10	NR	NR	Discovery ST (GE Healthcare)	12–18
Takesh et al, 2012 [24]	18F	250 MBq	QL	10	2	N	Siemens Biograph 6 (Siemens/CTI)	12(mean)
Wieder et al, 2017 [31]	11C	600–900 MBq	QL, Semi-QN	5	2	Y	Siemens Sensation 16 Biograph (Siemens)	24–38

^a^Time from injection to scan.

QL, qualitative; Semi-QN, semi-quantitative; N, no; Y, yes; NR, not reported.

### Quality assessment

The summary of the quality assessment is illustrated in Table 3. With regard to the patient selection domain, four studies [12, 13, 24, 30] were considered to have an unclear risk of bias because they did not explicitly mention whether patient recruitment was consecutive or not. High applicability concerns for patient selection domain were found in two studies because one study [13] only included patients showing a single lesion on BS, and the other [27] excluded patients with more than four metastatic bone lesions. In terms of the index test domain, there was high risk of bias in two studies [12, 24], as the interpretation of PET/CT was not blinded to the reference standard. There was no concern for applicability of the index test in all included studies. With regard to the reference standard domain, there was an unclear risk of bias in all studies except two [14, 25] because it was unclear whether the reference standard assessments were blinded to the index test for most studies. The risk of bias for the flow and timing domain was judged as high in five studies [12, 13, 25, 27, 29] because different reference standards were applied within these studies. In general, the quality of the currently available studies was considered reasonable, with 12 of the 14 studies satisfying at least four of the seven QUADAS-2 domains.

**Table 3:** QUADAS-2 risk of bias assessment.

Study references	Risk of bias				Applicability concerns		
	Patient selection	Index test	Reference standard	Flow and timing	Patient selection	Index test	Reference standard
Beheshti et al, 2010 [12]	Unclear	High	Unclear	High	Low	Low	Low
Evangelista et al, 2015 [22]	Low	Unclear	Unclear	Low	Low	Low	Low
Fuccio et al, 2010 [13]	Unclear	Unclear	Unclear	High	High	Low	Low
Garcia et al, 2015 [27]	Low	Low	Unclear	High	High	Low	Low
Huysse et al, 2017 [28]	Low	Low	Unclear	Low	Low	Low	Low
Kitajima et al, 2014 [23]	Low	Low	Unclear	Low	Low	Low	Low
Kitajima et al, 2017 [26]	Low	Low	Unclear	Low	Low	Low	Low
Langsteger et al, 2011 [14]	Low	Low	Low	Low	Low	Low	Low
McCarthy et al, 2011 [29]	Low	Low	Unclear	High	Low	Low	Low
Nanni et al, 2016 [25]	Low	Unclear	High	High	Low	Low	Low
Picchio et al, 2012 [21]	Low	Low	Unclear	Low	Low	Low	Low
Piccardo et al, 2014 [30]	Unclear	Unclear	Unclear	Low	Low	Low	Low
Takesh et al, 2012 [24]	Unclear	High	Unclear	Low	Low	Low	Low
Wieder et al, 2017 [31]	Low	Low	Unclear	Low	Low	Low	Low

### Diagnostic accuracy

**Fig 2:**
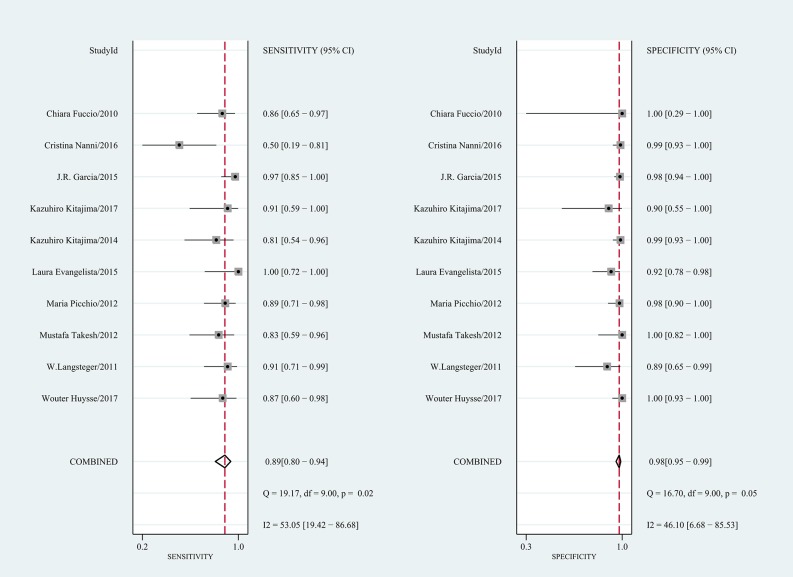
Forest plot of the pooled sensitivity and specificity of choline PET/CT on a per-patient basis.

**Fig 3:**
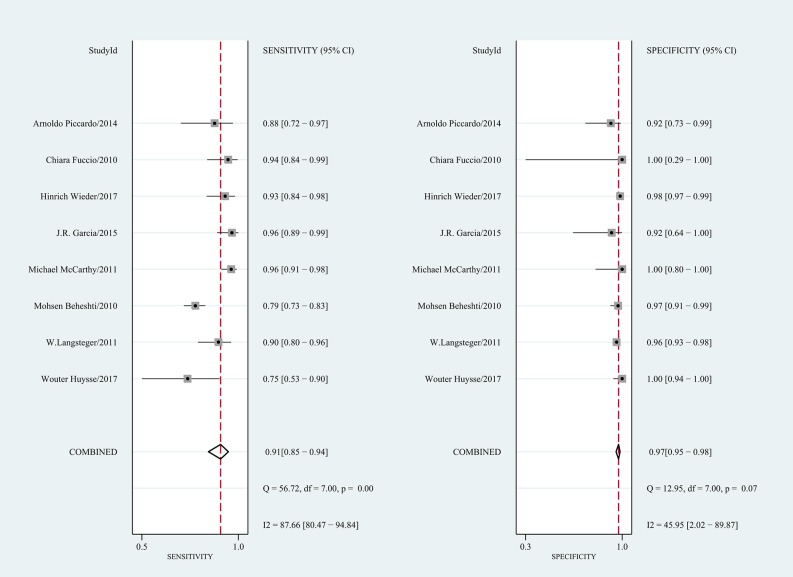
Forest plot of the pooled sensitivity and specificity of choline PET/CT on a per-lesion basis.

**Fig 4:**
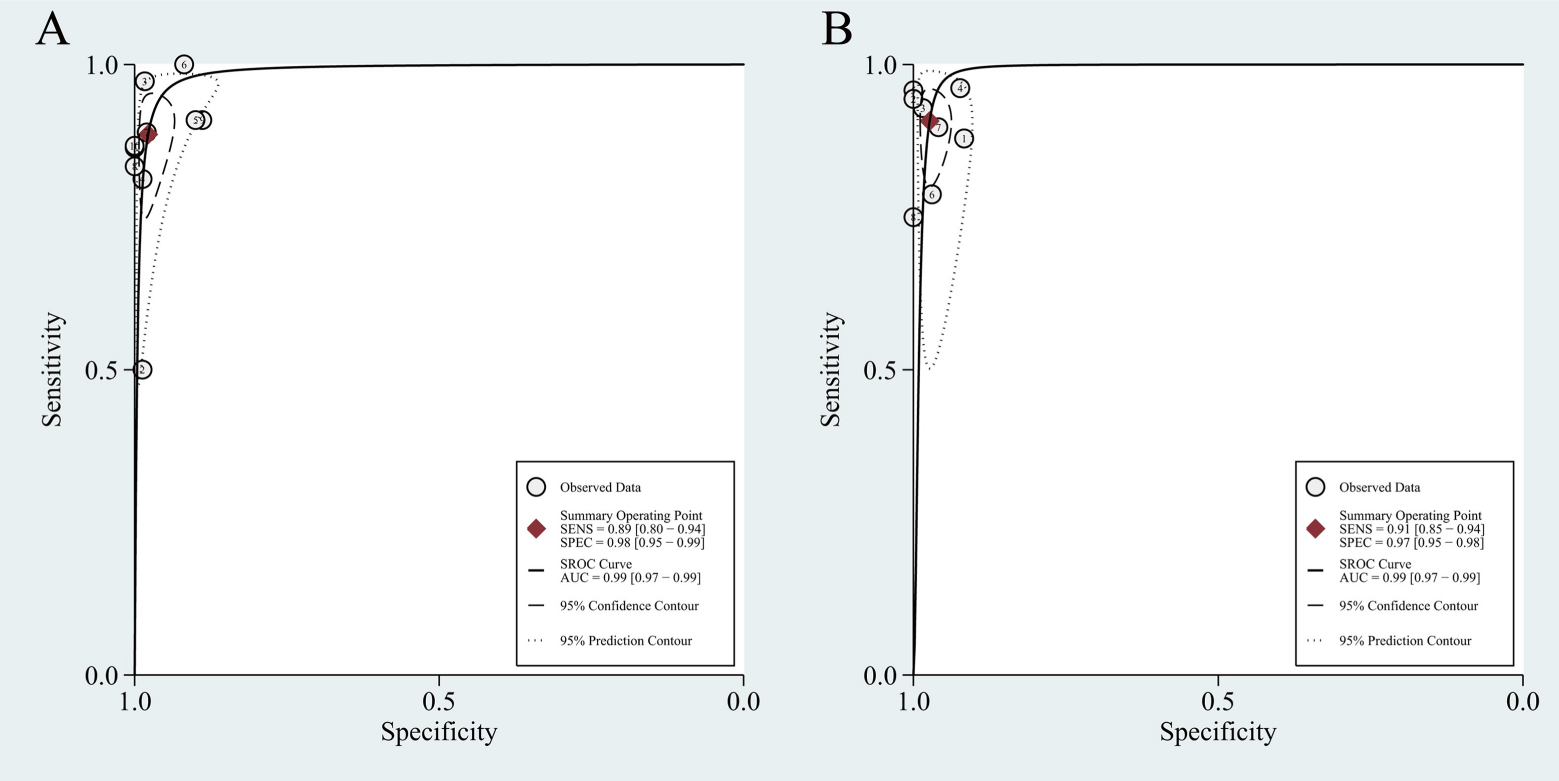
Hierarchical summary receiver operating characteristic curves for choline PET/CT on a per-patient basis (A) and on a per-lesion basis (B).

Table 4 shows the pooled results. On a per-patient basis, 10 studies involving 655 patients were included. The reported sensitivity and specificity of the included studies ranged from 50% to 100% and from 89% to 100%, respectively. For all 10 studies, the pooled sensitivity, specificity, and PLR, NLR, and DOR values were 0.89 (95% CI 0.80–0.94), 0.98 (95% CI 0.95–0.99), 40.4 (95% CI 19.7–82.6), 0.12 (95% CI 0.07–0.20), and 344 (95% CI 148–803), respectively. We recorded no threshold effect (Spearman correlation coefficient = 0.457; p = 0.184). The forest plot of the sensitivity and specificity also revealed the lack of a threshold effect (Fig 2). The heterogeneity was moderate in terms of specificity (Q = 16.70; p = 0.05; I^2^ = 46.10%); however, in terms of sensitivity, it was substantial (Q = 19.17; p = 0.02; I^2^ = 53.05%). On a per-lesion basis, 8 studies involving 472 patients with 1,619 lesions were included. The reported sensitivity and specificity ranged from 75% to 96% and from 92% to 100%, respectively. For all 8 studies, the pooled sensitivity, specificity, PLR, NLR, and DOR values were 0.91 (95% CI 0.85–0.94), 0.97 (95% CI 0.95–0.98), 34.1 (95% CI 20.0–58.1), 0.10 (95% CI 0.06–0.16), and 358 (95% CI 165–778), respectively (Table 4). No threshold effect was shown (Spearman correlation coefficient = 0.357; p = 0.385). The coupled forest plot of the sensitivity and specificity also indicated no threshold effect (Fig 3). The heterogeneity was substantial with regard to sensitivity (Q = 56.72; p = 0.00; I^2^ = 87.66%), and it was moderate with regard to specificity (Q = 12.95; p = 0.07; I^2^ = 45.95%). The HSROC curves are presented in Fig 4, and the area under the HSROC curve was 0.99 (95% CI 0.97–0.99) for per-patient analysis and 0.99 (95% CI 0.97–0.99) for per-lesion analysis.

**Table 4:** Pooled analysis of the diagnostic performance for choline PET-CT on a per-patient basis and on a per-lesion basis.

Data Type	Imaging Methods	number of studies	number of patients (lesions)	Sensitivity (95% CI)	Specificity (95% CI)	PLR (95% CI)	NLR (95% CI)	DOR (95% CI)	AUC
Patient based	11C/18F	10	655	0.89(0.80–0.94)	0.98(0.95–0.99)	40.4(19.7–82.6)	0.12(0.07–0.20)	344(148–803)	0.99(0.97–0.99)
	11C	6	466	0.87(0.74–0.94)	0.98(0.96–0.99)	50.3(22.5–112.8)	0.13(0.06–0.28)	376(120–1180)	0.99(0.97–0.99)
	18F	4	189	0.90(0.78–0.96)	0.97(0.85–1.00)	31.3(5.5–176.6)	0.10(0.05–0.23)	298(47–1893)	0.96(0.94–0.97)
Lesion based	11C/18F	8	472(1619)	0.91(0.85–0.94)	0.97(0.95–0.98)	34.1(20.0–58.1)	0.10(0.06–0.16)	358(165–778)	0.99(0.97–0.99)
	18F	5	221(1017)	0.88(0.78–0.93)	0.97(0.94–0.98)	25.3(15.3–41.6)	0.13(0.07–0.23)	196(96–401)	0.98(0.96–0.99)

CI, confidence intervals; PLR, positive likelihood ratio; NLR, negative likelihood ratio; DOR, diagnostic odds ratio; AUC, area under the HSROC curve; 11C, 11C-choline; 18F, 18F-choline.

### Exploration of heterogeneity

The results of the meta-regression analyses are shown in Table 5. On a per-patient basis, the clinical setting was likely the only source of study heterogeneity. Specifically, studies including only restaging PC patients reported a significantly higher specificity than those including only initial staging PC patients (0.99 vs. 0.91; p = 0); however, the pooled sensitivity estimates were not significantly different (0.87 vs. 0.95; p = 0.16). Upon analysis of the other covariates, study design, tracer, reference standard, diagnostic criteria and blinding to reference standard were not shown to be significant factors affecting the heterogeneity. We also performed a sensitivity analysis excluding a single study [25] that had a high risk of bias for the reference standard and showed a particularly low sensitivity of 0.50. The analysis yielded a lower degree of heterogeneity (I^2^ = 0 and 46.12 for sensitivity and specificity, respectively), with a sensitivity of 0.90 (95% CI 0.84–0.94) and a specificity of 0.98 (95% CI 0.94–0.99). We did not perform meta-regression analyses for per-lesion basis because of the limited number of included studies.

**Table 5:** Meta-regression analysis on a per-patient basis.

Covariates	Subgroup	Univariate analysis				Multivariate analysis	
		Sensitivity(95%CI)	p value	Specificity(95%CI)	p value	LRT χ^2^	p value
Study design	Prospective	0.88(0.79–0.97)	0.20	0.98(0.96–1.00)	0.31	0.06	0.97
	Retrospective	0.89(0.80–0.98)		0.98(0.95–1.00)			
Tracer	11C-choline	0.87(0.78–0.95)	0.11	0.98(0.97–1.00)	0.56	1.71	0.43
	18F-choline	0.91(0.82–1.00)		0.96(0.92–1.00)			
Clinical setting	Initial staging	0.95(0.85–1.00)	0.16	0.91(0.83–1.00)	0.00	6.55	0.04
	Restaging	0.87(0.80–0.94)		0.99(0.97–1.00)			
Reference standard	Histopathology/ clinical follow up	0.85(0.73–0.96)	0.05	0.99(0.97–1.00)	0.91	2.96	0.23
	Only clinical follow up	0.91(0.84–0.98)		0.96(0.93–0.99)			
Diagnostic criteria	Qualitative and semi-quantitative	0.86(0.74–0.97)	0.09	0.98(0.97–1.00)	0.49	1.23	0.54
	Qualitative	0.90(0.83–0.97)		0.97(0.94–1.00)			
Blinding	Yes	0.91(0.85–0.97)	0.66	0.98(0.96–1.00)	0.51	2.16	0.34
	No	0.83(0.71–0.95)		0.97(0.94–1.00)			

CI, confidence intervals; LRT, likelihood ratio test.

### Publication bias

**Fig 5:**
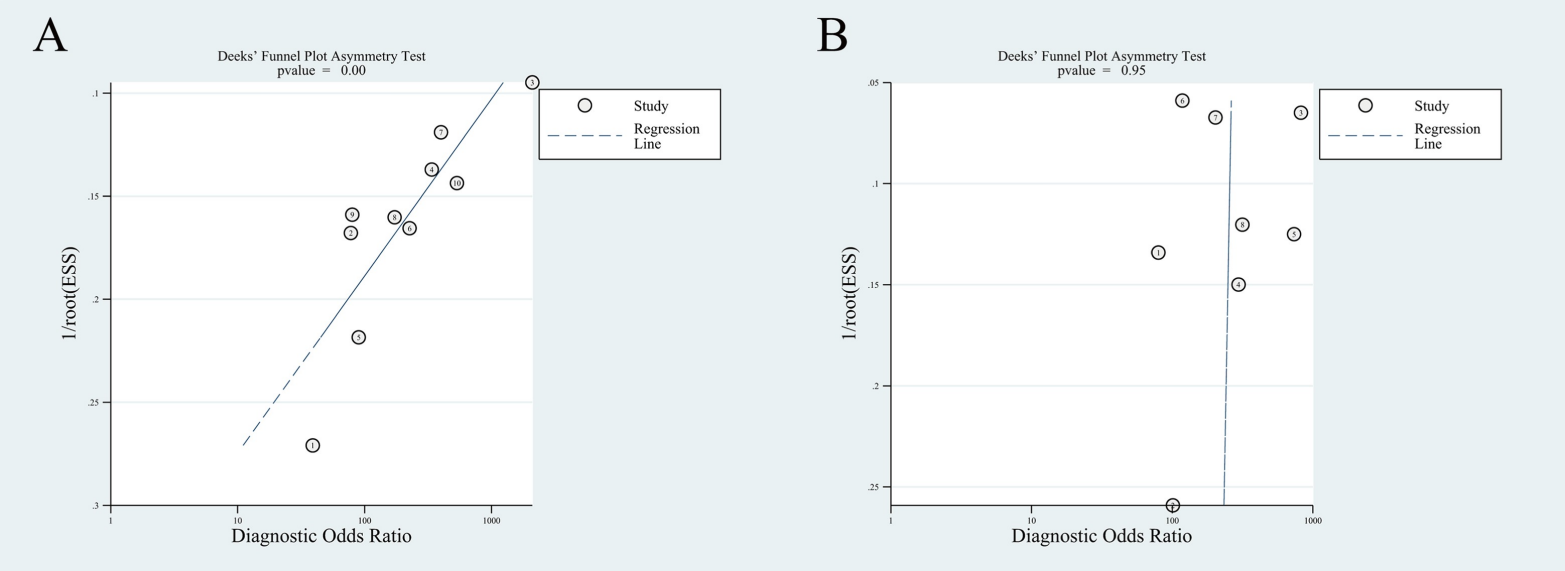
Deeks’ funnel plot test of choline PET/CT on a per-patient basis (A) and on a per-lesion basis (B).

The Deek’s funnel plot asymmetry test suggested the presence of publication bias for per-patient basis (p = 0), while no publication bias was found for per-lesion basis (p = 0.95) (Fig 5).

## Discussion

Previously, two meta-analyses have investigated the diagnostic accuracy of choline-PET/CT for detecting bone metastases in PC. Fanti et al. [15] conducted a meta-analysis of the literature published until December 2014 assessing 11C-choline PET/CT for its accuracy in the restaging of patients. He reported that in all eight studies the overall detection rate for bone metastases was 25%. Unfortunately, in only four studies the data of sensitivity and specificity for skeletal metastases were reported, and with very high heterogeneity. Thus, it is not wise to draw hasty conclusion of diagnostic accuracy from this meta-analyses. In a meta-analysis from 2014, Shen et al. [16] summarized the literature on choline PET/CT, MRI, SPECT, and BS in detecting bone metastases for PC. Shen indicated that MRI was better than choline PET/CT and BS on a per-patient basis, and choline PET/CT was better than BS and SPECT on a per-lesion analysis. However, this meta-analysis has substantial shortcomings in its quantitative data analysis. It summarized pairs of sensitivity and specificity into a single measure of diagnostic accuracy. Thus, important information is missing, and furthermore, the researchers did not assess the heterogeneity of patients from different settings or other study-specific covariates. To retain the two-dimensional character, we used the bivariate random effects regression model to synthesize data, which was more likely to be scientific. In this meta-analysis, we demonstrated that choline PET/CT performs well as a diagnostic modality for assessing skeletal metastases in PC, with an area under the HSROC curve of 0.99 both on a per-patient basis and on a per-lesion basis. Such adjacency of the area under the HSROC curve to 1 is a strong indicator of high diagnostic accuracy. According to our analysis, the pooled DOR values on a per-patient basis and on a per-lesion basis were respectively 344 and 358, also suggesting a high level of overall accuracy.

Currently, given its low cost, easy availability and large clinical experience, 99mTc-phosphonates BS (99mTc-BS) is the most widely used agent for assessing bone metastases in PC despite its well-known limited diagnostic performance [9]. A recent meta-analysis by Treglia discovered that the discordance rate was 10.9% between choline PET/CT and BS in detecting bone metastases in PC [32]. Treglia considered that discordant findings were likely related to the different mechanism of uptake of radioactive tracers. 99mTc-phosphonates accumulate in osteoplastic lesions, which are the response to bone destruction and are not tumor specific [8]. Thus, metastatic bone lesions are identified indirectly by 99mTc-BS. Conversely, choline is a substrate for the synthesis of phospholipids that are necessary for the formation of cell membranes [33, 34]. Accordingly, PET radiotracers such as 11C-choline or 18F-choline are supposed to target tumor cells directly [35]. In this study, choline PET/CT showed a pooled sensitivity of 89% and specificity of 98% to diagnose bone metastases in PC, which is superior to the reported performance of BS (i.e., pooled sensitivity and specificity of 71% and 91%, respectively) [36].

The optimum tracer for PET/CT in PC remains a matter of debate. Both 11C-choline and 18F-choline have been investigated. 11C-choline presents a shorter half-life (20 min) that makes its use limited to institutions with a cyclotron [37]. In contrast, 18F-choline is available to institutions without an onsite cyclotron because of its longer half-life (110 min), but the urinary excretion of 18F-choline is higher than 11C-choline, which may interfere with the interpretation of imaging findings in the pelvis [38, 39]. A previous review reported that 11C-choline and 18F-choline had similar diagnostic performance for malignant lesions in different clinical settings [40]. Our meta-analysis also found that both 11C-choline and 18F-choline PET/CT had excellent sensitivity (0.87 vs. 0.90) and specificity (0.98 vs. 0.97) for detecting bone metastases in PC patients. Our findings strengthen the current evidence for the use of PET/CT with 11C-choline or 18F-choline as tracers.

In the restaging setting, it is important to determine whether there is recurrent disease, lymph nodes or bone metastases, for the purpose of seeking an appropriate therapeutic planning [13]. Many researchers have demonstrated that choline PET/CT is useful for restaging PC, especially for detecting distant skeletal metastases. Fuccio et al. [41] detected a total of 30 bone lesions not revealed by BS in 18 of 123 restaging PC patients (14.6%) through 11C-choline PET/CT. Garcia et al. [27] found that choline PET/CT allowed early detection of bone metastases in 19.6% of restaging patients with negative BS results, thereby avoiding unnecessary treatment. Usefulness of choline PET/CT in initial staging setting is limited but encouraging. It was reported that choline PET/CT was not recommended for the initial diagnosis considering its low sensitivity in detecting primary lesions [42]. However, Evangelista’s study had emerged that 18F-choline PET/CT could accurately stage PC patients with an intermediate to high risk of systemic disease [22]. A prospective study [43] demonstrated that based on choline PET/CT results, the therapy plan was changed from curative intent to palliative care in 20% of staging patients. The application of choline PET/CT in initial staging of PC patients warrants further investigation.

We further examined the diagnostic accuracy by calculating the PLR and NLR, which were more clinically meaningful than the HSROC and DOR. A PLR greater than 10 or an NLR less than 0.1 provide convincing evidence to rule in or rule out disease [44]. Our study revealed that the pooled PLR values on a per-patient basis and on a per-lesion basis were 40.4 and 34.1, respectively, which were high enough to verify bone metastases. At the same time, the pooled NLR values on a per-patient basis and on a per-lesion basis were 0.12 and 0.10, respectively. Hence, a negative choline PET/CT result may be insufficient to exclude bone metastases in PC. Lower 11C-choline uptake was observed in osteoblastic metastases compared to osteolytic lesions [45, 46]. Beheshti et al. [12] confirmed that there was a significant correlation between tracer uptake and the density of malignant lesions with HU (Hounsfield unit) levels < 825 on CT. Besides, no choline uptake was detected in sclerotic bone lesions (with HU > 825). These performances, therefore, proves that the imaging may yield false-negative results. Moreover, it has been demonstrated that the PSA level significantly influenced the sensitivity of choline PET/CT [31, 47]. Choline PET/CT may not be routinely indicated in case the serum PSA level rises < 1 ng/ml. Reported data showed a variable detection rate according to PSA level, ranging from 36% if PSA at relapse was lower than 1 ng/ml to 73% if PSA was higher than 3 ng/ml [25, 48]. However, few papers included in this meta-analysis reported accurate data stratified by PSA values, which made it impossible to deliver a reasonable comparison. The recent introduction of gallium-68 prostate specific membrane antigen (Ga-68 PSMA) as a PET tracer might further improve results [49]. Ga-68 PSMA PET/CT has a detection rate of 50% and 68%, respectively for PSA levels < 0.5 ng/ml and 0.5–2 ng/ml [50]. However, its use is restricted due to limited availability and high costs.

There was significant heterogeneity among the included studies. The meta-regression analysis indicated that the clinical setting may be the only source of heterogeneity on a per-patient basis. Compared with studies including only initial staging patients, studies including only restaging patients showed a significantly higher specificity (0.99 vs. 0.91; p = 0) and a tendency for lower sensitivity (0.87 vs. 0.95; p = 0.16). We hypothesized that if patients have already received systemic therapies, the imaging features of malignant bone lesions may change. As mentioned above, Beheshti et al. [46] found that no choline uptake was detected in densely sclerotic bone lesions, almost all of which were observed in patients with hormone therapy. Another study [51] reported a significant reduction of choline uptake following androgen deprivation therapy in androgen-sensitive patients with recurrent PC. The presence of systemic therapies may cause false-negative PET/CT findings and affect diagnostic performance. However, Picchio et al. [21] documented that the accuracy of 11C-choline PET/CT for detecting skeletal metastasis in hormone-resistant patients did not significantly differ from patients who did not receive anti-androgenic treatment. A similar phenomenon was described by Kitajima1 et al [26]. In our study, we were unable to perform separate analyses based on the type of treatments, as the separate diagnostic performance values could not be extracted from the included studies. The impact of systemic therapies prior to choline PET/CT scanning remains unclear.

Among the included studies, only a few used “histopathology and clinical follow-up” as the gold standard, while most relied on clinical follow-up and conventional imaging modalities to confirm the existence of bone metastasis. Different reference standards might be an important source of heterogeneity, although our analysis suggested no significant difference among the subgroups. Furthermore, other covariates, including study design, tracer, diagnostic criteria and blinding to the reference standard, were not shown to be significant factors influencing the heterogeneity. However, in one study [25], all negative PET/CT scans were considered false negatives due to a PSA rise, except in two patients, who had a negative 24-month follow-up. This study showed an unusually inferior sensitivity of 0.50 and was considered to have a high risk of bias for the reference standard. Therefore, a sensitivity analysis was performed excluding this single study as a result, we achieved consistent diagnostic performance with substantially decreased heterogeneity, with a sensitivity of 0.90 (95% CI 0.84–0.94, I^2^ = 0) and a specificity of 0.98 (95% CI 0.94–0.99, I^2^ = 46.12).

This meta-analysis has several limitations [52]. First, the lack of a well-accepted gold standard may have affected the evaluation of choline PET/CT. The gold reference standard for any diagnostic study is histological confirmation of the findings. Nevertheless, in clinical practice, we could not perform a biopsy on each lesion. In this meta-analysis, we had to use “histopathology and/or clinical follow-up” as the suboptimal reference tests. Another limitation is that we detected publication bias on a per-patient basis. Generally, studies with desirable results may be more likely to be published than those with neutral or unfavorable results [53]. To minimize the possibility of publication bias, we searched the databases and reference lists of included articles again for further potential studies, but we could not obtain additional relevant publications. In addition, limiting the search to the English language and the exclusion of abstracts, case reports, letters, and comments may have also produced potential publication bias. Furthermore, the characteristics of the clinical variables among these selected studies, such as PSA level, technical parameters and measurements, were heterogeneous, making a stratified analysis for different risk groups impossible. Finally, caution is needed when applying our results because most of the included studies were from the United States and Europe, and only one Asian study with a relatively small sample size was included.

In conclusion, this systematic literature review and meta-analysis demonstrated that choline PET/CT had excellent sensitivity and specificity for the detection of bone metastasis in PC patients, both on a per-patient basis and on a per-lesion basis. However, a negative choline PET/CT result could not ensure the lack of bone metastasis.

## Supporting information

S1 File PRISMA 2009 checklist. (DOC)

S1 Table Primary data for per-patient basis. (XLSX)

S2 Table Primary data for per-lesion basis. (XLSX)

## References

[1] Ferlay J, Soerjomataram I, Dikshit R, Eser S, Mathers C, Rebelo M, et al Cancer incidence and mortality worldwide: sources, methods and major patterns in GLOBOCAN 2012. Int J Cancer. 2015;136(5):E359–86. 10.1002/ijc.29210 . 25220842

[2] Jemal A, Bray F, Center MM, Ferlay J, Ward E, Forman D. Global cancer statistics. CA Cancer J Clin. 2011;61(2):69–90. 10.3322/caac.20107 . 21296855

[3] Dotan ZA. Bone imaging in prostate cancer. Nat Clin Pract Urol. 2008;5(8):434–44. 10.1038/ncpuro1190 . 18682719

[4] Jemal A, Siegel R, Xu J, Ward E. Cancer statistics, 2010. CA Cancer J Clin. 2010;60(5):277–300. 10.3322/caac.20073 . 20610543

[5] Norgaard M, Jensen AO, Jacobsen JB, Cetin K, Fryzek JP, Sorensen HT. Skeletal related events, bone metastasis and survival of prostate cancer: a population based cohort study in Denmark (1999 to 2007). The Journal of urology. 2010;184(1):162–7. 10.1016/j.juro.2010.03.034 . 20483155

[6] Han M, Partin AW, Zahurak M, Piantadosi S, Epstein JI, Walsh PC. Biochemical (prostate specific antigen) recurrence probability following radical prostatectomy for clinically localized prostate cancer. The Journal of urology. 2003;169(2):517–23. 10.1097/01.ju.0000045749.90353.c7 . 12544300

[7] Cornford P, Bellmunt J, Bolla M, Briers E, De Santis M, Gross T, et al EAU-ESTRO-SIOG Guidelines on Prostate Cancer. Part II: Treatment of Relapsing, Metastatic, and Castration-Resistant Prostate Cancer. European urology. 2017;71(4):630–42. 10.1016/j.eururo.2016.08.002 . 27591931

[8] Beheshti M, Langsteger W, Fogelman I. Prostate cancer: role of SPECT and PET in imaging bone metastases. Seminars in nuclear medicine. 2009;39(6):396–407. 10.1053/j.semnuclmed.2009.05.003 . 19801219

[9] Bombardieri E, Setti L, Kirienko M, Antunovic L, Guglielmo P, Ciocia G. Which metabolic imaging, besides bone scan with 99mTc-phosphonates, for detecting and evaluating bone metastases in prostatic cancer patients? An open discussion. The quarterly journal of nuclear medicine and molecular imaging: official publication of the Italian Association of Nuclear Medicine. 2015;59(4):381–99. . 26337240

[10] Weber WA, Grosu AL, Czernin J. Technology Insight: advances in molecular imaging and an appraisal of PET/CT scanning. Nat Clin Pract Oncol. 2008;5(3):160–70. 10.1038/ncponc1041 . 18253106

[11] Kitajima K, Murphy RC, Nathan MA, Sugimura K. Update on positron emission tomography for imaging of prostate cancer. Int J Urol. 2014;21(1):12–23. 10.1111/iju.12250 . 23991644

[12] Beheshti M, Vali R, Waldenberger P, Fitz F, Nader M, Hammer J, et al The use of F-18 choline PET in the assessment of bone metastases in prostate cancer: correlation with morphological changes on CT. Molecular imaging and biology: MIB: the official publication of the Academy of Molecular Imaging. 2010;12(1):98–107. 10.1007/s11307-009-0239-7 . 19588206

[13] Fuccio C, Castellucci P, Schiavina R, Santi I, Allegri V, Pettinato V, et al Role of 11C-choline PET/CT in the restaging of prostate cancer patients showing a single lesion on bone scintigraphy. Annals of nuclear medicine. 2010;24(6):485–92. 10.1007/s12149-010-0390-x . 20544323

[14] Langsteger W, Balogova S, Huchet V, Beheshti M, Paycha F, Egrot C, et al Fluorocholine (18F) and sodium fluoride (18F) PET/CT in the detection of prostate cancer: prospective comparison of diagnostic performance determined by masked reading. The quarterly journal of nuclear medicine and molecular imaging: official publication of the Italian Association of Nuclear Medicine. 2011;55(4):448–57. . 21738117

[15] Fanti S, Minozzi S, Castellucci P, Balduzzi S, Herrmann K, Krause BJ, et al PET/CT with (11)C-choline for evaluation of prostate cancer patients with biochemical recurrence: meta-analysis and critical review of available data. European journal of nuclear medicine and molecular imaging. 2016;43(1):55–69. 10.1007/s00259-015-3202-7 . 26450693

[16] Shen G, Deng H, Hu S, Jia Z. Comparison of choline-PET/CT, MRI, SPECT, and bone scintigraphy in the diagnosis of bone metastases in patients with prostate cancer: a meta-analysis. Skeletal radiology. 2014;43(11):1503–13. 10.1007/s00256-014-1903-9 . 24841276

[17] Hamza TH, van Houwelingen HC, Stijnen T. The binomial distribution of meta-analysis was preferred to model within-study variability. Journal of clinical epidemiology. 2008;61(1):41–51. 10.1016/j.jclinepi.2007.03.016 . 18083461

[18] Whiting PF, Rutjes AW, Westwood ME, Mallett S, Deeks JJ, Reitsma JB, et al QUADAS-2: a revised tool for the quality assessment of diagnostic accuracy studies. Ann Intern Med. 2011;155(8):529–36. 10.7326/0003-4819-155-8-201110180-00009 . 22007046

[19] Deeks JJ, Macaskill P, Irwig L. The performance of tests of publication bias and other sample size effects in systematic reviews of diagnostic test accuracy was assessed. Journal of clinical epidemiology. 2005;58(9):882–93. 10.1016/j.jclinepi.2005.01.016 . 16085191

[20] Higgins JP, Thompson SG. Quantifying heterogeneity in a meta-analysis. Stat Med. 2002;21(11):1539–58. 10.1002/sim.1186 . 12111919

[21] Picchio M, Spinapolice EG, Fallanca F, Crivellaro C, Giovacchini G, Gianolli L, et al [11C]Choline PET/CT detection of bone metastases in patients with PSA progression after primary treatment for prostate cancer: comparison with bone scintigraphy. European journal of nuclear medicine and molecular imaging. 2012;39(1):13–26. 10.1007/s00259-011-1920-z . 21932120

[22] Evangelista L, Cimitan M, Zattoni F, Guttilla A, Zattoni F, Saladini G. Comparison between conventional imaging (abdominal-pelvic computed tomography and bone scan) and [(18)F]choline positron emission tomography/computed tomography imaging for the initial staging of patients with intermediate- tohigh-risk prostate cancer: A retrospective analysis. Scandinavian journal of urology. 2015;49(5):345–53. 10.3109/21681805.2015.1005665 . 25649494

[23] Kitajima K, Murphy RC, Nathan MA, Froemming AT, Hagen CE, Takahashi N, et al Detection of recurrent prostate cancer after radical prostatectomy: comparison of 11C-choline PET/CT with pelvic multiparametric MR imaging with endorectal coil. Journal of nuclear medicine: official publication, Society of Nuclear Medicine. 2014;55(2):223–32. 10.2967/jnumed.113.123018 . 24434294

[24] Takesh M, Odat Allh K, Adams S, Zechmann C. Diagnostic Role of (18)F-FECH-PET/CT Compared with Bone Scan in Evaluating the Prostate Cancer Patients Referring with Biochemical Recurrence. ISRN oncology. 2012;2012:815234 10.5402/2012/815234 ; PubMed Central PMCID: PMC3515921. 23251818 PMC3515921

[25] Nanni C, Zanoni L, Pultrone C, Schiavina R, Brunocilla E, Lodi F, et al (18)F-FACBC (anti1-amino-3-(18)F-fluorocyclobutane-1-carboxylic acid) versus (11)C-choline PET/CT in prostate cancer relapse: results of a prospective trial. European journal of nuclear medicine and molecular imaging. 2016;43(9):1601–10. 10.1007/s00259-016-3329-1 . 26960562

[26] Kitajima K, Fukushima K, Yamamoto S, Kato T, Odawara S, Takaki H, et al Diagnostic performance of (11)C-choline PET/CT and bone scintigraphy in the detection of bone metastases in patients with prostate cancer. Nagoya J Med Sci. 2017;79(3):387–99. doi: 10.18999/nagjms.79.3.387 ; PubMed Central PMCID: PMCPMC5577024. 28878443 10.18999/nagjms.79.3.387 PMC5577024

[27] Garcia JR, Moreno C, Valls E, Cozar P, Bassa P, Soler M, et al [Diagnostic performance of bone scintigraphy and (11)C-Choline PET/CT in the detection of bone metastases in patients with biochemical recurrence of prostate cancer]. Revista espanola de medicina nuclear e imagen molecular. 2015;34(3):155–61. 10.1016/j.remn.2014.08.001 . 25443648

[28] Huysse W, Lecouvet F, Castellucci P, Ost P, Lambrecht V, Artigas C, et al Prospective Comparison of F-18 Choline PET/CT Scan Versus Axial MRI for Detecting Bone Metastasis in Biochemically Relapsed Prostate Cancer Patients. Diagnostics. 2017;7(4). 10.3390/diagnostics7040056 ; PubMed Central PMCID: PMC5745392. 29039785 PMC5745392

[29] McCarthy M, Siew T, Campbell A, Lenzo N, Spry N, Vivian J, et al (1)(8)F-Fluoromethylcholine (FCH) PET imaging in patients with castration-resistant prostate cancer: prospective comparison with standard imaging. European journal of nuclear medicine and molecular imaging. 2011;38(1):14–22. 10.1007/s00259-010-1579-x . 20862471

[30] Piccardo A, Paparo F, Piccazzo R, Naseri M, Ricci P, Marziano A, et al Value of fused 18F-Choline-PET/MRI to evaluate prostate cancer relapse in patients showing biochemical recurrence after EBRT: preliminary results. BioMed research international. 2014;2014:103718 10.1155/2014/103718 ; PubMed Central PMCID: PMC4022120. 24877053 PMC4022120

[31] Wieder H, Beer AJ, Holzapfel K, Henninger M, Maurer T, Schwarzenboeck S, et al 11C-choline PET/CT and whole-body MRI including diffusion-weighted imaging for patients with recurrent prostate cancer. Oncotarget. 2017;8(39):66516–27. doi: 10.18632/oncotarget.16227 ; PubMed Central PMCID: PMC5630432. 29029532 10.18632/oncotarget.16227 PMC5630432

[32] Treglia G, Vigneri C, Sadeghi R, Evangelista L, Ceriani L, Giovanella L. Discordance rate between radiolabelled choline PET/CT and bone scintigraphy in detecting bone metastases in patients with prostate cancer: a meta-analysis. Clinical and Translational Imaging. 2015;3(2):133–40. 10.1007/s40336-015-0107-1

[33] Zeisel SH. Dietary choline: biochemistry, physiology, and pharmacology. Annu Rev Nutr. 1981;1:95–121. 10.1146/annurev.nu.01.070181.000523 . 6764726

[34] Ramirez de Molina A, Rodriguez-Gonzalez A, Gutierrez R, Martinez-Pineiro L, Sanchez J, Bonilla F, et al Overexpression of choline kinase is a frequent feature in human tumor-derived cell lines and in lung, prostate, and colorectal human cancers. Biochemical and biophysical research communications. 2002;296(3):580–3. . 12176020 10.1016/s0006-291x(02)00920-8

[35] Wondergem M, van der Zant FM, van der Ploeg T, Knol RJ. A literature review of 18F-fluoride PET/CT and 18F-choline or 11C-choline PET/CT for detection of bone metastases in patients with prostate cancer. Nuclear medicine communications. 2013;34(10):935–45. 10.1097/MNM.0b013e328364918a . 23903557

[36] Cheng X, Li Y, Xu Z, Bao L, Li D, Wang J. Comparison of 18F-FDG PET/CT with bone scintigraphy for detection of bone metastasis: a meta-analysis. Acta radiologica. 2011;52(7):779–87. 10.1258/ar.2011.110115 . 21712464

[37] Hara T, Kosaka N, Kishi H. PET imaging of prostate cancer using carbon-11-choline. Journal of nuclear medicine: official publication, Society of Nuclear Medicine. 1998;39(6):990–5. . 9627331

[38] Cimitan M, Bortolus R, Morassut S, Canzonieri V, Garbeglio A, Baresic T, et al [18F]fluorocholine PET/CT imaging for the detection of recurrent prostate cancer at PSA relapse: experience in 100 consecutive patients. European journal of nuclear medicine and molecular imaging. 2006;33(12):1387–98. 10.1007/s00259-006-0150-2 . 16865395

[39] Kwee SA, Wei H, Sesterhenn I, Yun D, Coel MN. Localization of primary prostate cancer with dual-phase 18F-fluorocholine PET. Journal of nuclear medicine: official publication, Society of Nuclear Medicine. 2006;47(2):262–9. . 16455632

[40] Jadvar H. Prostate cancer: PET with 18F-FDG, 18F- or 11C-acetate, and 18F- or 11C-choline. Journal of nuclear medicine: official publication, Society of Nuclear Medicine. 2011;52(1):81–9. 10.2967/jnumed.110.077941 ; PubMed Central PMCID: PMCPMC3012154. 21149473 PMC3012154

[41] Fuccio C, Castellucci P, Schiavina R, Guidalotti PL, Gavaruzzi G, Montini GC, et al Role of C-11-choline PET/CT in the re-staging of prostate cancer patients with biochemical relapse and negative results at bone scintigraphy. European journal of radiology. 2012;81(8):E893–E6. 10.1016/j.ejrad.2012.04.027 PubMed PMID: WOS:000307126200014. 22621862

[42] Fuccio C, Rubello D, Castellucci P, Marzola MC, Fanti S. Choline PET/CT for prostate cancer: main clinical applications. European journal of radiology. 2011;80(2):e50–6. 10.1016/j.ejrad.2010.07.023 . 20800404

[43] Kjolhede H, Ahlgren G, Almquist H, Liedberg F, Lyttkens K, Ohlsson T, et al Combined 18F-fluorocholine and 18F-fluoride positron emission tomography/computed tomography imaging for staging of high-risk prostate cancer. BJU Int. 2012;110(10):1501–6. 10.1111/j.1464-410X.2012.11123.x . 22502982

[44] Deeks JJ. Systematic reviews in health care: Systematic reviews of evaluations of diagnostic and screening tests. BMJ. 2001;323(7305):157–62. ; PubMed Central PMCID: PMCPMC1120791. 11463691 10.1136/bmj.323.7305.157 PMC1120791

[45] Ceci F, Castellucci P, Graziani T, Schiavina R, Chondrogiannis S, Bonfiglioli R, et al 11C-choline PET/CT identifies osteoblastic and osteolytic lesions in patients with metastatic prostate cancer. Clinical nuclear medicine. 2015;40(5):e265–70. 10.1097/RLU.0000000000000783 . 25783519

[46] Beheshti M, Vali R, Waldenberger P, Fitz F, Nader M, Loidl W, et al Detection of bone metastases in patients with prostate cancer by 18F fluorocholine and 18F fluoride PET-CT: a comparative study. European journal of nuclear medicine and molecular imaging. 2008;35(10):1766–74. 10.1007/s00259-008-0788-z . 18465129

[47] Castellucci P, Fuccio C, Nanni C, Santi I, Rizzello A, Lodi F, et al Influence of trigger PSA and PSA kinetics on 11C-Choline PET/CT detection rate in patients with biochemical relapse after radical prostatectomy. Journal of nuclear medicine: official publication, Society of Nuclear Medicine. 2009;50(9):1394–400. 10.2967/jnumed.108.061507 . 19690023

[48] Krause BJ, Souvatzoglou M, Tuncel M, Herrmann K, Buck AK, Praus C, et al The detection rate of [11C]choline-PET/CT depends on the serum PSA-value in patients with biochemical recurrence of prostate cancer. European journal of nuclear medicine and molecular imaging. 2008;35(1):18–23. 10.1007/s00259-007-0581-4 . 17891394

[49] Afshar-Oromieh A, Zechmann CM, Malcher A, Eder M, Eisenhut M, Linhart HG, et al Comparison of PET imaging with a (68)Ga-labelled PSMA ligand and (18)F-choline-based PET/CT for the diagnosis of recurrent prostate cancer. European journal of nuclear medicine and molecular imaging. 2014;41(1):11–20. 10.1007/s00259-013-2525-5 ; PubMed Central PMCID: PMC3843747. 24072344 PMC3843747

[50] Evangelista L, Briganti A, Fanti S, Joniau S, Reske S, Schiavina R, et al New Clinical Indications for (18)F/(11)C-choline, New Tracers for Positron Emission Tomography and a Promising Hybrid Device for Prostate Cancer Staging: A Systematic Review of the Literature. European urology. 2016;70(1):161–75. 10.1016/j.eururo.2016.01.029 . 26850970

[51] Fuccio C, Schiavina R, Castellucci P, Rubello D, Martorana G, Celli M, et al Androgen deprivation therapy influences the uptake of 11C-choline in patients with recurrent prostate cancer: the preliminary results of a sequential PET/CT study. European journal of nuclear medicine and molecular imaging. 2011;38(11):1985–9. 10.1007/s00259-011-1867-0 . 21732105

[52] Whiting PF, Rutjes AW, Westwood ME, Mallett S, Group Q-S. A systematic review classifies sources of bias and variation in diagnostic test accuracy studies. Journal of clinical epidemiology. 2013;66(10):1093–104. 10.1016/j.jclinepi.2013.05.014 . 23958378

[53] Guyatt GH, Oxman AD, Montori V, Vist G, Kunz R, Brozek J, et al GRADE guidelines: 5. Rating the quality of evidence—publication bias. Journal of clinical epidemiology. 2011;64(12):1277–82. 10.1016/j.jclinepi.2011.01.011 . 21802904

